# Placental MRI Predicts Fetal Oxygenation and Growth Rates in Sheep and Human Pregnancy

**DOI:** 10.1002/advs.202203738

**Published:** 2022-08-28

**Authors:** Dimitra Flouri, Jack R. T. Darby, Stacey L. Holman, Steven K. S. Cho, Catherine G. Dimasi, Sunthara R. Perumal, Sebastien Ourselin, Rosalind Aughwane, Nada Mufti, Christopher K. Macgowan, Mike Seed, Anna L. David, Andrew Melbourne, Janna L. Morrison

**Affiliations:** ^1^ School of Biomedical Engineering and Imaging Sciences King's College London London SE1 7EU UK; ^2^ Department of Medical Physics and Biomedical Engineering University College London London WC1E 6BT UK; ^3^ Early Origins of Adult Health Research Group Health and Biomedical Innovation UniSA Clinical and Health Sciences University of South Australia Adelaide SA 5001 Australia; ^4^ Department of Physiology The Hospital for Sick Children University of Toronto Toronto ON M5G 1X8 Canada; ^5^ South Australian Health & Medical Research Institute Preclinical Imaging & Research Laboratories Adelaide SA 5001 Australia; ^6^ Elizabeth Garrett Anderson Institute for Women's Health University College London London WC1E 6AU UK; ^7^ Division of Translational Medicine The Hospital for Sick Children University of Toronto Toronto ON M5G 1X8 Canada; ^8^ Department of Medical Biophysics University of Toronto Toronto ON M5S 1A1 Canada; ^9^ Department of Paediatrics Division of Cardiology The Hospital for Sick Children University of Toronto Toronto ON M5G 1X8 Canada; ^10^ Department of Diagnostic Imaging The Hospital for Sick Children University of Toronto Toronto ON M5G 1X8 Canada; ^11^ NIHR Biomedical Research Centre University College London Hospitals London W1T 7DN UK

**Keywords:** fetal development, fetal growth restriction, fetal hypoxia, oxygenation, placental dysfunction, relaxometry, sheep

## Abstract

Magnetic resonance imaging (MRI) assessment of fetal blood oxygen saturation (SO_2_) can transform the clinical management of high‐risk pregnancies affected by fetal growth restriction (FGR). Here, a novel MRI method assesses the feasibility of identifying normally grown and FGR fetuses in sheep and is then applied to humans. MRI scans are performed in pregnant ewes at 110 and 140 days (term = 150d) gestation and in pregnant women at 28^+3^ ± 2^+5^ weeks to measure feto‐placental SO_2_. Birth weight is collected and, in sheep, fetal blood SO_2_ is measured with a blood gas analyzer (BGA). Fetal arterial SO_2_ measured by BGA predicts fetal birth weight in sheep and distinguishes between fetuses that are normally grown, small for gestational age, and FGR. MRI feto‐placental SO_2_ in late gestation is related to fetal blood SO_2_ measured by BGA and body weight. In sheep, MRI feto‐placental SO_2_ in mid‐gestation is related to fetal SO_2_ later in gestation. MRI feto‐placental SO_2_ distinguishes between normally grown and FGR fetuses, as well as distinguishing FGR fetuses with and without normal Doppler in humans. Thus, a multi‐compartment placental MRI model detects low placental SO_2_ and distinguishes between small hypoxemic fetuses and normally grown fetuses.

## Graphical abstract

The authors use a sheep model of human pregnancy to validate magnetic resonance imaging (MRI) fetal oxygenation measurements with invasive blood gas‐samples. They present evidence that this method is predictive of fetal growth rate in sheep and human pregnancies. These findings may lead to development of novel MRI‐based diagnostic tool for tracking fetal oxygenation in pregnancies complicated by placental insufficiency.

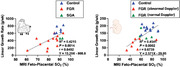

## 1 Introduction

The fetus grows and develops within the mother's uterus, which presents a challenge to a clinician's ability to monitor fetal well‐being. However, understanding the health of the placenta, the interface between the mother and the fetus that facilitates the transport of oxygen and nutrients from the mother to the fetus, informs the clinician of fetal health. Placental insufficiency is associated with the reduced transfer of oxygen and nutrients, preventing the fetus from reaching its genetic growth potential^[^ ^1^ ^]^ and resulting in fetal growth restriction (FGR). Concerningly, FGR not only predisposes an infant to preterm delivery and a greater risk of both morbidity or death at or around the time of birth, but also predisposes surviving neonates to coronary artery disease, obesity, diabetes, and hypertension in later life, a phenomenon known as developmental programming.^[^ ^2^, ^3^, ^4^ ^]^

Some fetuses are detected as being small (< 10^th^ centile estimated fetal weight (EFW); small for gestational age; SGA) but many do not experience adverse pregnancy outcomes expected with a reduction in nutrient or oxygen supply. Thus, they are considered constitutionally small rather than FGR and to have reached their genetic growth potential. Whilst these SGA fetuses are not at higher risk of adverse pregnancy outcomes, they are difficult to distinguish from those at‐risk FGR babies and thus may enter a program of increased surveillance and intervention such as induction of labor aimed at mitigating against poor pregnancy outcomes. As stillbirth in late gestation occurs at a higher rate in FGR pregnancies,^[^ ^5^ ^]^ the clinical aim is to best time their delivery so that they can be cared for outside the womb. Unfortunately, some FGR fetuses are not detected through routine antenatal care leading to an increased rate of stillbirth, while SGA fetuses are misdiagnosed as FGR leading to unnecessary preterm and early term delivery. Thus, differentiating the FGR fetus from a healthy SGA fetus remains an unmet challenge.

FGR affects up to 8% of pregnancies worldwide^[^ ^5^ ^]^ and is diagnosed when a fetus is SGA, has a small abdominal circumference (AC), abnormal growth trajectory, or abnormal Doppler parameters in the umbilical artery and/or middle cerebral artery.^[^ ^6^, ^7^, ^8^, ^9^ ^]^ When invasive cordocentesis testing of fetal oxygen saturation (SO_2_) is performed, fetuses with an AC < 5^th^ centile have both lower PO_2_ and SO_2_.^[^ ^10^, ^11^ ^]^ However, such invasive tests have the potential to cause miscarriage, preterm premature rupture of the membranes (PROM), and subsequent preterm birth and are generally not performed in this setting. Current clinical management of FGR, therefore, includes sonographic serial assessment of fetal size, amniotic fluid, and fetal movements, as well as the measurement of fetal and maternal uterine artery and fetal arterial/venous vascular resistance and waveforms, derived from Doppler ultrasound to indicate the severity of the placental insufficiency.^[^ ^1^, ^12^ ^]^ However, these parameters only assess the fetal circulatory response to placental oxygen and nutrient supply and are unable to directly assess fetal oxygenation. Thus, fetal blood oxygenation status is not a parameter that is currently used to detect and characterize FGR in humans. Non‐invasive assessment of fetal blood SO_2_ would have a significant clinical impact, especially in the differential diagnosis and management of pregnancies affected by FGR and their differentiation from SGA.

Blood flow velocity in the placenta as assessed by Doppler ultrasound of the uterine and umbilical arteries has limited predictive value for FGR and provides limited information regarding fetal oxygenation status.^[^ ^13^, ^14^ ^]^ However, the blood flow and permeability properties of the placenta can also be explored using magnetic resonance imaging (MRI). MRI is sensitive to a much broader range of tissue properties than ultrasound and the distinct signals coming from different placental tissue compartments can be measured by combining acquisition types and forming a multi‐compartment model of the placental tissue.^[^ ^15^ ^]^ MRI techniques exploit the dependence of MR relaxation times such as *T*_1_, *T*_2_, and *T*_2_∗, on the level of oxyhemoglobin in blood^[^ ^16^, ^17^ ^]^ and, non‐invasive MR methods have been used to estimate SO_2_ of blood in fetal blood vessels,^[^ ^18^, ^19^, ^20^, ^21^ ^]^ and the placenta itself.^[^ ^15^, ^22^, ^23^, ^24^, ^25^, ^26^ ^]^ A disadvantage of targeted placental scans is the overlap between maternal and fetal blood within any given imaging region. Here, we overcome this issue by using a new imaging methodology mechanism (Diffusion‐rElaxation Combined Imaging for Detailed Placental Evaluation; DECIDE) that separates fetal and maternal circulations in the placenta.^[^ ^15^ ^]^ Our previous work in human pregnancy has shown that the measurement of placental function using DECIDE, especially feto‐placental SO_2_ is correlated with disease severity in early onset FGR.^[^ ^27^ ^]^

The pregnant sheep is a robust preclinical model for pregnancy research that has provided an evidence base for clinical translation of intervention strategies to improve pregnancy outcomes.^[^ ^28^ ^]^ Both the size of the ewe and her fetus are comparable to a human mother and neonate, and thus potential clinical imaging and surgical technologies can be tested in this setting. The sheep placenta serves the same functional purpose as in the human, although its structure is somewhat different.^[^ ^29^ ^]^ However, in both species, the placenta maintains the oxygen and nutritional status of the fetus, and complications of placentation can lead to FGR in similar patterns. A key advantage of using sheep as a preclinical model of human pregnancy is that fetal surgery can be performed to implant catheters into the fetal arteries to allow for sampling of fetal blood. Daily sampling results in a profile of fetal arterial partial pressure of oxygen (PO_2_) and SO_2_ across late gestation using a conventional blood gas analyzer (BGA).

**Figure 1:**
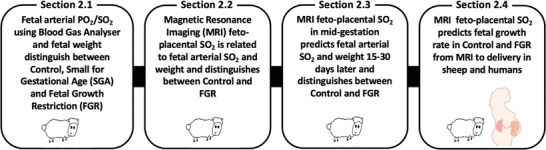
Diagram illustrating the four sections of this study.

Here, we investigate a non‐invasive MRI technique with the clinical potential to measure fetal blood SO_2_ within the placenta, and we study the relationship between this measure of feto‐placental SO_2_ and fetal growth in sheep and human fetuses (**Figure** **1**). We propose that the clinical application of this technique in pregnancies complicated by FGR, where the non‐invasive assessment of fetal oxygenation status is not possible, would be valuable in pregnancy management and improving maternal and fetal outcomes.

## 2 Results and Interpretation

### 2.1 Fetal PO_2_ and SO_2_ are Determinants of Fetal Weight and Relative Brain Weight in Late Gestation

Preclinical studies show that the fetal brain can respond to changes in fetal arterial PO_2_. In the setting of acute hypoxemia, a decrease in PO_2_ sensed by the carotid bodies initiates a neuroendocrine and cardiovascular response.^[^ ^30^, ^31^ ^]^ For example, the fetus has increased distribution of cardiac output to the brain in response to hypoxia,^[^ ^30^ ^]^ which is thought to lead to a common characteristic of FGR known as “brain sparing” where the ratio of brain to body weight is greater than expected for the small body weight.^[^ ^32^ ^]^ In humans, this “brain sparing” effect is often observed sonographically as increased diastolic flow velocity in the middle cerebral arteries.^[^ ^33^, ^34^, ^35^, ^36^ ^]^ Although this is a fetal physiological response to changes in oxygenation, it is associated in the long term with neurodevelopmental pathology.^[^ ^33^ ^]^ Preclinical models of FGR in which the fetus is chronically hypoxemic (low arterial PO_2_)^[^ ^10^, ^37^ ^]^ reveal that the extent of some fetal adaptations is related to the degree of chronic hypoxemia.^[^ ^38^, ^39^ ^]^ However, in preclinical models and human FGR studies of chronic fetal hypoxemia, the extent of the redistribution of cardiac output is diminished compared with that seen in acute hypoxemia.^[^ ^40^, ^41^, ^42^ ^]^ This may be explained by the lack of carotid body response to chronic hypoxemia in FGR.^[^ ^31^ ^]^ For these reasons, fetal PO_2_ represents an important independent marker of fetal health status.

**Figure 2:**
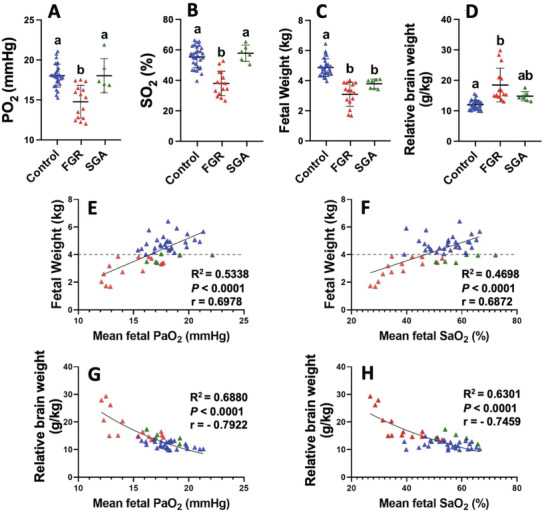
Relationships between mean gestational fetal arterial a) PO_2_ and B) SO_2_ with postmortem fetal weight and relative brain weight (a hallmark of FGR) at 140d (term = 150d) across control normally grown sheep fetuses (blue triangles, *n* = 29) and those that are chronically hypoxemic with FGR (< 10^th^ centile) due to carunclectomy pre‐conception leading to placental insufficiency (red triangles, *n* = 15). Dashed line represents the 10^th^ centile for control pregnancies (4.0 kg). A group of control but SGA fetuses that are normoxemic and < 10^th^ centile estimated fetal weight is included (green triangles, n = 7, not included in regression analysis). Data presented as individual data points with superimposed mean ±SD where each point represents a different fetus. Data was analyzed by a one‐way ANOVA with a A—D) Bonferroni correction for multiple comparisons, and either E,F) linear (E,F) or G,H) exponential regressions. Alphabetic nomenclature represents statistical differences between groups whereby different letters represent groups that are statistically different from each other. *p <* 0.05 was considered statistically significant for all analyzes. FGR, fetal growth restriction (< 10^th^ centile, CX group, and hypoxemic); SGA, small for gestational age (< 10^th^ centile, control group, and normoxemic).

MRI techniques are sensitive to SO_2_ but not PO_2_ (due to the magnetic differences between oxy‐ and deoxy‐hemoglobin, rather than to oxygen content per se). Here we use our preclinical sheep model to show that FGR fetuses from ewes that had undergone carunclectomy (CX) surgery prior to conception to reduce the potential sites for placentation (*n* = 15), all had a body weight less than the 10^th^ centile of controls and most had a mean gestation PO_2_ less than 17 mmHg, a previously defined cut off for chronic hypoxemia in sheep fetuses that are subject to FGR (**Figure** **2**A–D; red triangles).^[^ ^39^, ^43^ ^]^ Within our cohort of control ewe pregnancies, we identified 7 fetuses with a low estimated fetal weight that had both a normal PO_2_ and SO_2_ across late gestation to 140 days gestation (10^th^ centile for the control group was 4.0 kg). These fetuses were then defined as SGA (Figure 2A–D; green triangles). The control normally grown, and SGA groups exhibited similar PO_2_ (Figure 2A; *p* > 0.9999) and SO_2_ (Figure 2B; *p* > 0.9999) but different fetal weight (Figure 2C; *p* = 0.0006). The FGR and SGA fetuses resulted in different PO_2_ (*p* = 0.0009) and SO_2_ (*P* < 0.0001) values but similar fetal weights. However, the FGR group had a higher relative brain weight compared to control normally grown fetuses (Figure 2D; *p* < 0.0001), whereas the relative brain weight of the SGA group was not different from that of control normally grown fetuses (Figure 2D). Importantly, in the control SGA fetuses, PO_2_ and SO_2_ can distinguish between a fetus with increased relative brain weight (FGR) and an SGA fetus with a normal relative brain weight (Figure 2H, *p* < 0.0001). While there is a relationship between PO_2_ and both fetal weight and relative brain weight in late gestation (140d, term = 150d; Figure 2E,G), there is also a strong relationship between fetal mean gestation SO_2_ and fetal body weight and relative brain weight at 140d gestation (term, 150d; Figure 2F,H). We also found that the mean gestational *Hb* (*Hb*_control_ = 95.6 ± 10.4 mm Hg, *Hb*_FGR_ = 110.3 ± 23.3 mm Hg, *P* = 0.0394) and mean gestational *Hct* (*Hct*_control_ = 28.1 ± 3.2%, *Hct*_FGR_ = 32.5 ± 6.8%, *P* = 0.03856) were higher in FGR than control fetuses. Thus, we set out to determine if it is possible to measure fetal SO_2_ noninvasively in the placenta and relate this to fetal oxygenation, and fetal weight.

### 2.2 MRI of Feto‐Placental Oxygen Saturation is Related to Fetal Arterial Oxygenation and Weight at the Time of the MRI Scan

MRI measurements of T_2_ are sensitive to oxygen saturation and in vitro measurements have shown that the Luz‐Meiboom physical model allows a one‐to‐one conversion between SO_2_ and *T*_2_ values.^[^ ^19^ ^]^ Measurement of a pure sample of fetal blood *T*_2_ depends on stable blood flow conditions, a fetus that does not move during the acquisition and specific vessel prescriptions^[^ ^19^ ^]^ whilst the downstream measurement of fetal blood *T*_2_ is itself only a surrogate marker for placental function. However, this approach can be used to measure placental oxygen consumption.^[^ ^18^ ^]^ Measuring the placenta itself is important for understanding the function of the organ and can be advantageous since placental motion artifacts are less severe compared to the free‐floating moving fetus.^[^ ^24^, ^44^, ^45^ ^]^ However, within any given region of the placenta the measured MRI signal will contain merged information from the independent maternal and fetal blood circulations, thus an alternative model is required when estimating a pure fetal blood *T*_2_ value in this way.

**Figure 3:**
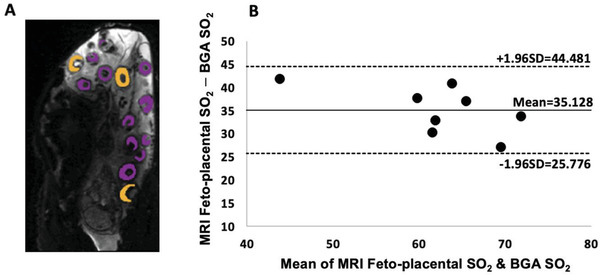
A) Example of placentome segmentation, the color corresponds to different placentome types.^[^ ^43^ ^]^ B) Bland–Altman plot comparing MRI feto‐placental oxygenation saturation (SO_2_) against reference blood gas analyzer (BGA) measured fetal descending aorta (DAo) blood SO_2_ sampled during the MRI scan whilst the ewes were under general anesthesia. The y‐axis shows the difference in fetal MRI estimates of oxygen saturation minus reference BGA oxygen saturation. Solid line indicates the mean and dashed lines indicate the 95% confidence interval.

Here, we use an image‐based technique named DECIDE, that separates the fetal and maternal circulations with an approach that is relatively rapid without the need for cardiac or respiratory gating. This allows the fetal blood *T*_2_ to be estimated from a region of placental tissue containing both maternal and fetal blood. In this study, we have assumed that the maternal blood and fetal blood are intra‐capillary but that the maternal blood has higher oxygen saturation than the fetal blood,^[^ ^15^ ^]^ which therefore allows for the discrimination of the maternal and fetal blood pools. Example placentome segmentations are shown in **Figure** **3**A.^[^ ^45^ ^]^ A Bland‐Altman plot comparing the MRI feto‐placental SO_2_ measurements against simultaneous BGA measures of fetal oxygenation measurements from fetal descending aorta (DAo) shows good agreement between MRI feto‐placental SO_2_ and gold‐standard blood gas samples (Figure 3B). The proposed model measures the oxygenation of fetal blood in the placenta, and we showed that the value is higher than the oxygenation of the blood sample from the fetal DAo measured with a BGA, a vessel that contains oxygenated blood from the placenta mixed with the less oxygenated venous blood. These differences in the oxygenation of MRI feto‐placental SO_2_ and fetal DAo SO_2_ result in a consistent bias of 35% (Figure 3B). The 95% limits of agreement across all animals ranged from 25.8% to 44.5%. Thus, our MRI‐derived feto‐placental oxygenation results are consistent with the intricacies of fetal circulation and what is known about oxygen distribution in the fetus.^[^ ^19^, ^20^, ^46^, ^47^, ^48^ ^]^

**Figure 4:**
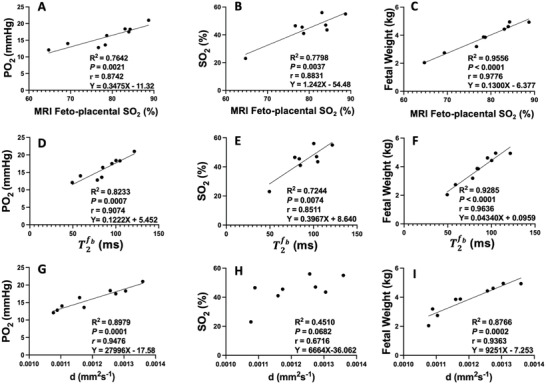
Relationships between the observed MRI feto‐placental oxygen saturation (SO_2_), feto‐placental blood relaxation time ($T_{2}^{f_{b}}$), and diffusivity (*d*) parameters estimated from the MRI model and the mean gestational fetal descending aorta (DAo) partial pressure of oxygen (PO_2_), average fetal DAo oxygen saturation (SO_2_) and fetal weight at postmortem the next day. Linear regression lines are depicted by black lines.

In nine animals, we measured blood gases daily to calculate mean gestational PO_2_ and SO_2_ in late gestation, performed the MRI and the next day collected fetal body and brain weight at post mortem. This allowed us to show that there is a strong relationship between MRI measures of feto‐placental SO_2_, diffusivity, and fetal blood volume in the placenta and conventional measures of fetal PO_2_ and SO_2_ across the previous 30 days as well as fetal weight (**Figure** **4**A–C). A significant linear relationship between MRI feto‐placental SO_2_ (*n* = 10) and BGA derived mean gestational fetal arterial SO_2_ was observed (*Y* = 1.24X–54.48, *R* ^2^ = 0.78, *r* = 0.88, *p* < 0.0001; Figure 4C).

The MRI model that we have developed estimates other properties of the placental structure and function. The diffusivity parameter (*d* reflects the microenvironment of the placenta based on the diffusion of water molecules. A more restricted or hindered diffusion environment is likely correlated to poorer oxygen transfer. A very strong positive linear correlation was observed between *d* and PO_2_, (*r* = 0.9476, *p* = 0.0001; Figure 4G). This is indicative of a restricted cellular environment or a denser cell space that therefore impedes the oxygen transfer between maternal and fetal blood pools. Another important finding is the strong linear correlation of *d* with fetal weight (Figure 4I). This result indicates that the lower the diffusivity in the placenta, the smaller the fetus. This observation is in line with previous studies on the human placenta.^[^ ^8^, ^49^ ^]^

The fetal blood volume fraction (*f*) can be thought of as the estimated percentage of the fetal blood volume within a voxel. Interestingly, *f* did not change between control and FGR placentas or with an increase in gestational age (**Table** **1**). However, we observed a strong negative linear correlation between *f* and the mean PO_2_ (Y = −20.20X + 22.69, R^2^ = 0.5697, r = −0.7548, p = 0.0187) and the average DAo SO_2_ (Y = −67.77X + 66.01, R^2^ = 0.533, r = −0.7301, p = 0.0397). This may be linked to an overgrowth of the fetal tissue of the placenta relative to maternal tissue resulting in eversion of placentomes and a higher prevalence of type C and D in response to chronic hypoxemia that is observed in the carunclectomy model of FGR.^[^ ^29^ ^]^ The maternal blood volume fraction (*v*) can be thought of as the estimated percentage of the maternal blood volume within a voxel and was not significantly different between groups (Table 1).

**Table 1:** Average MRI parameters derived over all singleton pregnancies at two gestational ages for all control normally grown and FGR placentomes. Results are presented as mean ± standard deviation. Significant differences between the two groups at the two gestational ages are shown in bold (*p <* 0.05). The parameters presented are MRI feto‐placental oxygen saturation (SO_2_), feto‐placental blood relaxation time$\left(T_{2}^{f_{b}}\right)$, trophoblast apparent diffusivity (*d*), feto‐placental blood volume fraction (*f*), maternal blood volume fraction (*v*), pseudo‐diffusivity *d*, fractional anisotropy (FA), and perfusion fraction estimated from intravoxel incoherent motion model (*f_IVIM_*)

MRI parameter	109–111d GA		139–141d GA		Effect of Group	Effect of GA	Interaction between group and GA
	Control (*n* = 10)	FGR (*n* = 13)	Control (*n* = 10)	FGR (*n* = 13)	*p*‐value	*p*‐value	*p*‐value
MRI feto‐placental SO_2_ [%]	89.85 ± 19.21	80.24 ± 8.13	85.68 ± 29.22	75.81 ± 5.96^a)^, ^b)^	**0.00012**	**0.01857**	0.908054
$T_{2}^{f_{b}}$ [ms]	125.81 ± 35.86	92.85 ± 24.41^a)^	110.10 ± 48.88^b)^	77.77 ± 16.39^a)^	**<0.0001**	**0.00646**	0.98152
*d* [mm^2^ s^−1^]	0.00159 ± 5e−4^b)^	0.00130 ± 2e−3^a)^, ^b)^	0.00126 ± 5e−4	0.00114 ± 6e−4^a)^, ^b)^	**0.00273**	**0.00047**	**0.04415**
*f* ***(no units)***	0.399 ± 0.095	0.427 ± 0.158	0.249 ± 0.061	0.380 ± 0.083	0.1844	0.0946	0.2727
*v* *(no units)*	0.291 ± 0.156	0.277 ± 0.218	0.244 ± 0.088	0.171 ± 0.088	0.694	0.205	0.802
d* (mm^2^ s^−1^)	0.014 ± 0.0021	0.021 ± 0.022	0.035 ± 0.023^b)^	0.043 ± 0.019^b)^	0.08872	**0.0083**	0.85523
FA (all b‐values)	0.485 ± 0.21	0.489 ± 0.125	0.642 ± 0.25^b)^	0.586 ± 0.196	0.6959	**0.0162**	0.4607
*f*_IVIM_ (no units)	0.240 ± 0.029^a)^, ^b)^	0.213 ± 0.024^a)^, ^b)^	0.295 ± 0.065	0.238 ± 0.049	**0.00096**	**<0.0001**	**0.0471**

^a)^ Effect of group

^b)^ Effect of age.

The fractional anisotropy (FA) is a frequently used index to measure diffusion anisotropy from images generated using diffusion tensor imaging (DTI).^[^ ^50^ ^]^ FA may provide unique insight into tissue microstructure. A statistically significant increase in FA was observed between 109–111 days and 139–141 days gestation (Table 1). Sheep placental development most likely causes changes in the relationship between maternal and placental villi and thus MRI may be sensitive to these changes that are otherwise only be seen with histopathology investigation.^[^ ^44^ ^]^ More complex measurements of FA have been performed in other tissues such as the brain^[^ ^51^, ^52^ ^]^ and may be appropriate to explore in the placenta. For example, a potential approach is to use orientation distribution functions,^[^ ^53^ ^]^ which might reveal differences in the complexity of fetal villi.

The data presented in this section show that the 139–140d gestation MRI measures of SO_2_ relate to the oxygenation level of the fetus over the previous 30 days as well as fetal weight the following day. The next step is to determine if MRI measures collected more than 30 days earlier in gestation can predict later mean gestational PO_2_ and SO_2_ and fetal weight.

### 2.3 MRI Measurement of Feto‐Placental Oxygen Saturation at Mid‐Gestation is Predictive of Placental Development and Fetal Growth later in Gestation

We have shown that invasive measurements of fetal oxygenation discriminate between control normally grown, FGR and SGA sheep fetuses. The relationship with PO_2_ is important as it provides a physiological link to the cascade of fetal responses following placental insufficiency‐induced FGR. In comparison, the relationship with SO_2_ is also essential because of its ubiquitous clinical role in monitoring patient wellbeing. We have also shown that MRI can be used to measure the oxygen saturation of fetal blood in the placenta and that this correlates strongly with invasive measurements from the fetus. In this section, we investigate the predictive power of early measurement of feto‐placental oxygen with later measurements from invasive fetal blood sampling.

**Figure 5. A):**
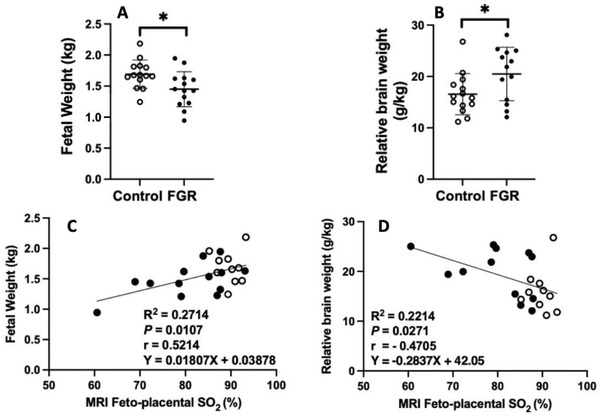
Fetal weight, B) relative brain weight, and C,D) their relationship with MRI feto‐placental SO_2_ as measured using MRI data at 110 days gestation. Normally grown control fetuses, unfilled circles; FGR fetuses, filled circles. Data analyzed by unpaired Student's *t*‐test or linear regression. Black line depicts a significant relationship between XY factors. *, *p <* 0.05. FGR, fetal growth restriction. Fetal weight and brain volume were determined from MRI images and converted to weight.

**Figure 6:**
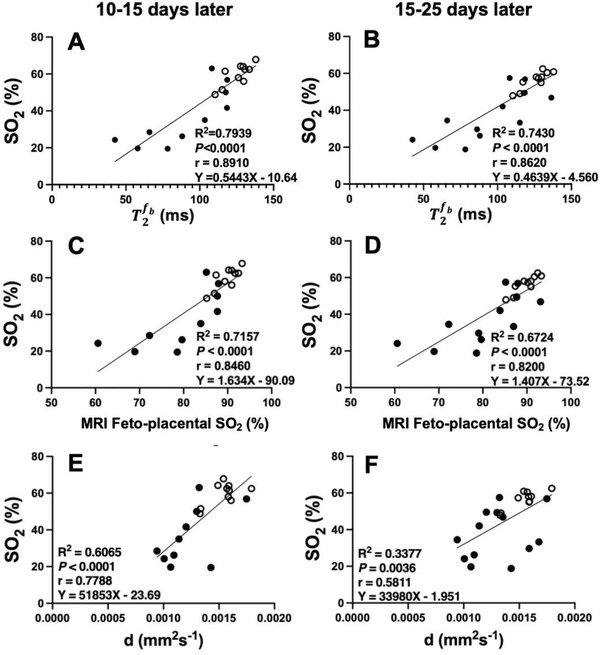
$T_{2}^{f_{b}}$, SO_2_ and *d* at 110 days gestation is predictive of fetal SO_2_ as measured by conventional blood gas analyzer 10–15 and 15–25 days later. Normally grown control fetuses, unfilled circles; FGR, fetal growth restriction fetuses, filled circles.

We used MR imaging data acquired at 110d gestation to show that fetal weight and relative brain volume (converted to weight) are lower in the FGR compared to the control normally grown fetuses (**Figure** **5**A,B). Furthermore, MRI feto‐placental SO_2_ is strongly related to fetal weight and relative brain weight at the time of the MRI scan in sheep (Figure 5C,D). We then use this data to determine if MRI feto‐placental SO_2_ can be used to predict gold‐standard invasive measurements of fetal SO_2_ in the DAo at 10–15 days and then at 15–25 days after the MRI. There is a moderate correlation between fetal weight at 110d gestation and fetal SO_2_ in the DAo 10–15 and 15–25 days later (**Figure** **6**C,D) as well as future body weight at delivery (140d, term = 150d; Figure 4C). Our MRI model of placental function is thus predictive of the long‐term trajectory of fetal blood oxygenation. Importantly, this suggests that a single MRI scan in mid‐gestation may have value in estimating future fetal outcomes in early‐onset FGR.^[^ ^28^ ^]^ As not all fetuses survived to post mortem at 140d gestation, we were unable to classify them as SGA versus FGR based on the criteria set out in Section 2.1. Here classification is by treatment group (control, no carunclectomy surgery, and FGR, preconception carunclectomy surgery). As such, we have not separated SGA from our FGR group. Given that chronic fetal hypoxemia is linked to poor short‐ and long‐term offspring outcomes, the use of these techniques may allow for earlier more accurate detection of at‐risk fetuses with enough time to customize both in utero and later life ex utero treatment plans for subsequent risk factors.

### 2.4 MRI Measurement of Feto‐Placental Oxygen Saturation is Predictive of Growth Rate in Sheep and Human Fetuses

Section 2.3 described the predictive value of mid‐gestation MRI for measuring growth and oxygenation status in later gestation. In the sheep arm of this study, we show a strong relationship between MRI feto‐placental SO_2_ at a single mid‐gestation time point and the trajectory of fetal oxygenation later in gestation. Our previous work in human pregnancy demonstrated the clinical potential for placental MRI, especially for measuring feto‐placental SO_2_, which was shown to correlate with disease severity in early onset FGR.^[^ ^27^ ^]^ In this section, we present the similarity in results from our sheep model of FGR pregnancy with those from FGR human pregnancy further supporting the potential value of this technique to clinical care of affected pregnancies.

**Figure 7:**
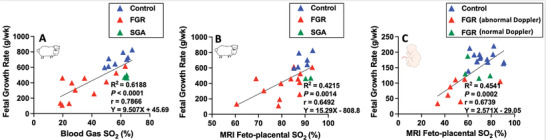
Both mean gestation fetal SO_2_ measured using a blood gas analyzer and MRI feto‐placental oxygen saturation (SO_2_) at 110 days gestation are positively related to the fetal growth rate (the increase in fetal weight from the time of MRI to tissue collection 13–31d later; A,B) in sheep. The same relationships exist in C) humans with MRI at 28 weeks of gestation and fetal weight growth rate from MRI to delivery up to 12 weeks later.

Measurement of fetal SO_2_ via conventional, invasive sampling for measurement on a BGA is predictive of fetal growth rate in sheep pregnancy (**Figure** **7**A). Our non‐invasive MRI measurement maintains this strong linear correlation. Measurement of oxygen status is predictive of the growth rate of the fetus in both sheep and human pregnancy (Figure 7B,C). Re‐analyzing the human data from Aughwane et al.^[^ ^27^ ^]^ shows that non‐invasive MRI measurement is also highly predictive of fetal growth rate (Figure 7C). Irrespective of gestational age, control fetuses have a higher MRI feto‐placental SO_2_ (73.7 ± 9.4%) than early‐onset FGR fetuses with abnormal Doppler ultrasound (55.9 ± 15.6%; *p* = 0.0009) but not those with normal Doppler ultrasound (66.5 ± 8.6%; *p* = 0.561). Thus, fetuses that have been classified as early‐onset FGR based on ultrasound estimates of fetal weight and Doppler information may be distinguished into two groups based on MRI feto‐placental SO_2_. This data shows the utility of MRI in measuring feto‐placental SO_2_ and predicting fetal weight as a marker of growth with the ability to identify FGR fetuses with different risk profiles. Close observation using serial ultrasound assessment of these early‐onset FGR fetuses would be standard of care. The introduction of a mid‐gestation MRI (as we performed in our pregnant sheep model of FGR) may lead to an evidence base to distinguish those SGA fetuses at risk of an adverse pregnancy outcome due to a reduction in oxygen and nutrient supply.

## 3 Discussion

We have shown that multi‐compartment modeling of non‐invasive placental MRI can be used to estimate the SO_2_ of feto‐placental blood in both sheep and human pregnancy. Firstly, we described the broad phenotype of our model of early‐onset FGR and the relationships between fetal weight and oxygenation status (Section 2.1). We then went on to present evidence that MRI markers of placental function correlate strongly with simultaneous gold standard fetal blood gas data (as measured by a conventional BGA) and fetal weight (Section 2.2). Next, we used our sheep model to demonstrate that these MR imaging techniques can be applied at one point in mid‐gestation to predict future fetal oxygenation (Section 2.3). Finally, we showed that not only are the measures obtained from these techniques predictive of future fetal growth rate and relative brain weight but that this relationship remains when noninvasively applied to human pregnancies (Section 2.4). Thus, evidence from sheep and human pregnancies shows that placental targeted MRI represents a valid means to assess fetal oxygenation with clear clinical utility in the setting of FGR.

Single‐compartment, mono‐exponential, models of placental tissue are widespread in the literature.^[^ ^43^, ^49^, ^54^, ^55^, ^56^ ^]^ However, while we have shown that *T*_2_ and *d* measures are an indicator of oxygen concentration,^[^ ^44^ ^]^ mono‐exponential decay models do not allow for separation between the maternal and fetal circulations and thus their interpretation is more challenging. When choosing a model, the user must trade‐off between acquisition simplicity and interpretability depending on the application.^[^ ^57^ ^]^ Here, we made use of DECIDE, a new technique for separating fetal and maternal circulations with an approach that is relatively rapid without the need for gating, and our MRI‐derived feto‐placental oxygenation results are consistent with what is known about oxygen distribution in the fetus.^[^ ^9^ ^]^ Nonetheless, the model makes several assumptions. We have assumed in the sheep that the maternal blood and fetal blood are intra‐capillary but that the maternal blood stays at high oxygen saturation compared to the fetal blood.^[^ ^15^ ^]^ This is unlikely to be completely true, especially in those FGR ewes where there is an increased oxygen gradient between ewe and fetus. It is, therefore, possible that there may be a small overlap between low‐saturation maternal venous blood and high‐saturation fetal venous blood where, although they are not physically mixing, would be interpreted by the model as being from the same source. Nonetheless, the high correlation of our results suggests that our assumption of separation is an effective first pass approximation and this supports the use of multi‐compartment models of the placenta more generally. This assumption could be relaxed in future models of placental function. For higher values of diffusion, the sensitivity of the MRI model used in this study becomes lower, thus separation of diffusion coefficients for maternal and fetal blood is not possible. It is also possible that information leveraged from diffusion tensor imaging could add information about blood delivery and further guide this separation especially as applied here to the sheep placenta and may make it possible to distinguish further flow information about the maternal and fetal blood. In addition, for the conversion of $T_{2}^{f_{b}}$ to fetal SO_2_ (Equation (1)), we have used an equation based upon in vitro human blood measurements.^[^ ^22^ ^]^ As discussed in^[^ ^18^ ^]^ there is a number of differences between the species that could affect the *T*_2_ relaxation times such as the fetal Hct during late gestation (human ≈48%, sheep ≈30%), red blood cell size (human‐large, sheep‐small,^[^ ^59^ ^]^ core body temperature (human‐37 °C, sheep‐39 °C), as well as any differences in blood protein composition. The fetal sheep *T*_2_ measurements made at 3T are lower in magnitude than at 1.5T for a given blood SO_2_ because of the magnetic field strength. However, previous work has calibrated blood *T*_2_ and SO_2_ at each field strength so that the derivation of SO_2_ values is not biased by differences in the field‐dependent range of *T*_2_ times.

Our study comprises data from two time points in mid‐ and late‐ gestation. We were unable to perform paired MRI studies in all animals because not all fetuses survived, either due to severe FGR or fetal demise during the period between the MRI scans. A further limitation is that fetal SO_2_ measurements in different vessels of the fetus were not possible in this study. We did not sample blood directly from the umbilical vein, which would have been the most highly saturated blood arriving in the fetus from the placenta. Rather, we sampled blood from the DAo, which is comprised of a mixture of oxygenated blood from the placenta and less oxygenated venous blood. Nonetheless, it remains an important validation step and is consistent with MRI measurements of fetal oxygenation obtained using different techniques.^[^ ^19^ ^]^ Indeed, the combination of the techniques utilized herein and those used to measure SO_2_ within the fetal circulation represents an interesting avenue for further work. Importantly, the correlation that we found holds across gestation allowing the non‐invasive tracking of fetal blood SO_2_ within the placenta. The experimental setting with surgery and anesthesia did not appear to have a major impact on placental oxygen and consumption. Data from ewes exposed to isoflurane anesthetic and humans not exposed to isoflurane showed a strong correlation between the MRI feto‐placenta SO_2_ and fetal weight in both cases. In addition, placental oxygen consumption measured with MRI was similar in both species despite only the sheep being exposed to isoflurane.^[^ ^18^ ^]^ Although we included a cohort of human pregnancies that contained normal and early‐onset FGR pregnancies and we were able to use our MRI model to separate these groups based on MRI feto‐placental MRI, we did not have a group of SGA human pregnancies to study. Such a comparison should be performed in future studies as this study highlights a potential key benefit of the technique described herein which is to differentiate between normoxemic appropriately grown, normoxemic SGA, and hypoxemic FGR human fetuses. Thus, this technique holds promise to better detect the at‐risk FGR fetus.

## 4 Conclusion

Our work supports the use of multi‐compartment placental MRI in human pregnancy. We have demonstrated a link between MRI‐derived measures of feto‐placental oxygenation and gold‐standard invasive blood gas measurements. This supports the possibility of using multi‐compartment MRI for the differential diagnosis of FGR and longitudinal non‐invasive tracking of feto‐placental SO_2_ in pregnancies with placental insufficiency. The use of advanced MRI to measure the physiological properties of the fetus holds much promise but it is only with validation in preclinical models that it will reach clinical acceptance. The establishment of more complex models of placental structure and function will help us to improve our understanding of this most fundamental of human organs.

## 5 Experimental Section

### Animal Data

**Figure 8:**
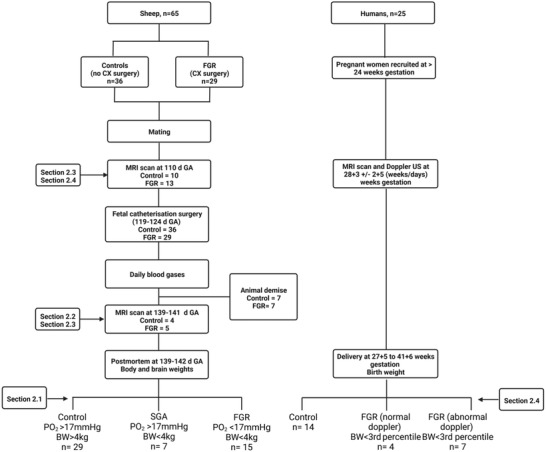
Data flow diagram illustrating the number of animals and subjects used in each measurement.

The study and all animal handling procedures were approved by the Animal Ethics Committee of the South Australian Health and Medical Research Institute (SAHMRI, SAM389.19) and abide by the Australian Code of Practice for the Care and Use of Animals for Scientific Purposes developed by the National Health and Medical Research Council and followed the ARRIVE guidelines.^[^ ^60^ ^]^ Ewes were sourced from the SAHMRI farm (Burra, South Australia, Australia) and housed in an indoor facility with a constant ambient temperature of 20–22 °C and a 12‐h light/dark cycle (see **Figure** **8**). Ewes were housed in individual pens in view of other sheep and had ad libitum access to food and water. All investigators understood the ethical principles outlined in Grundy et al^[^ ^61^ ^]^ and the principles of the 3Rs.^[^ ^62^ ^]^

### Carunclectomy Surgery

Non‐pregnant Merino ewes (*n* = 29) were assigned to have most of their endometrial caruncles (sites of potential future placentation) removed via carunclectomy (CX) as previously described.^[^ ^39^, ^63^ ^]^ Conception following carunclectomy resulted in the development of growth‐restricted sheep pregnancies.^[^ ^38^, ^64^, ^65^ ^]^ General anesthesia (induction: intravenous diazepam [0.3 mg kg^−1^] and ketamine [7 mg kg^−1^]); maintenance: 2% isoflurane in O2). Ewes received an intramuscular injection of antibiotics (3.5 ml of Duplocillin (150 mg ml^−1^ procaine penicillin and 112.5 mg ml^−1^ benzathine penicillin; Norbrook Laboratories Ltd., Gisborne, Australia) and 2 ml of 125 mg ml^−1^ Dihydrostreptomycin (Sigma, St Louis, MO, USA)) on the day of and for 3d following surgery. All ewes received the analgesic meloxicam (0.5 mg kg^−1^, subcutaneously) on the day before surgery and 24 h later.^[^ ^66^ ^]^ After a minimum 10‐week recovery period, CX ewes and control ewes (no previous surgery; *n* = 36) entered a mating program (see Figure 8).

### Fetal Catheterization Surgery

Time‐mated singleton‐bearing Merino ewes (*n* = 65; Control, *n* = 36; CX, *n* = 29) were transported from the farm at ≈90 days gestation (term = 150d). All ewes underwent fetal catheterization surgery at 109–124 days gestation under aseptic conditions as previously described.^[^ ^39^, ^67^, ^68^ ^]^ General anesthesia was induced and maintained as per the CX surgery. Vascular catheters were implanted into the maternal jugular vein, fetal femoral vein, and fetal femoral artery (tip in the DAo at the level of the renal artery) as well as the amniotic cavity. The fetus was returned to the uterus, which was sutured closed. A small incision was made in the ewe's flank, allowing exteriorization of the fetal catheters. After the closure of the abdomen, ewes received an intramuscular injection of antibiotics (3.5 ml of Duplocillin [150 mg ml^−1^ procaine penicillin and 112.5 mg ml^−1^ benzathine penicillin; Norbrook Laboratories Ltd., Gisborne, Australia) and 2 ml of 125 mg ml^−1^ Dihydrostreptomycin (Sigma, St Louis, MO, USA]) for 3 days following surgery. Fetuses received an intramuscular injection of 1 ml of Duplocillin and 1 ml of Dihydrostreptomycin during surgery. All ewes received the analgesic meloxicam (0.5 mg kg^−1^, subcutaneously) on the day before surgery and 24 h later.^[^ ^66^ ^]^ Each fetus received intra‐amniotic antibiotics (500 mg; sodium ampicillin, Commonwealth Serum Laboratories) for 4d post‐surgery.

After fetal surgery, fetal arterial blood samples were collected daily to monitor fetal health by measuring the partial pressure of oxygen (PaO_2_), partial pressure of carbon dioxide (PaCO_2_), oxygen saturation (SO_2_), pH, hemoglobin, hematocrit, base excess, and lactate with a RAPIDPOINT 500 BGA (Siemens Healthineers, Erlangen, Germany), calibrated for sheep blood. This data was divided into three periods depending on the number of blood gas samples collected after the 1st MRI at 110 days gestation to 10–15 days later (*n* = 23). Of these, 17 fetuses also had further blood gas sampling from 15–25 days after the 1^st^ MRI. The remaining ten fetuses continued until the 2^nd^ MRI (137–139 days gestation) and postmortem the next day. Means for each period were calculated for regression analysis.

### MR Imaging

At 109–111 days gestation, ewes underwent MRI scans (Control, *n* = 13; CX, *n* = 15; total *n* = 28). A subset of ewes had a further MRI scan at 139–141d GA (Control, *n* = 4; CX, *n* = 5; total *n* = 9). General anesthesia was induced and maintained as per CX surgery. The ewe was then positioned on its left side for the duration of the scan and ventilated to create normal fetal oxygenation (respiratory rate 16–18; ≈1L O_2_ and 5L air [109–111d GA] or ≈2L O_2_ and 4L air [139–141d GA]).^[^ ^69^ ^]^ Maternal heart rate and SO_2_ were measured using an MRI‐compatible SO_2_/heart rate monitor (Nonin Medical Inc, Plymouth, USA) and measurements were continuously recorded using LabChart 7 (ADInstruments, Castle Hill, Australia).^[^ ^19^, ^70^ ^]^ MRI was performed on a 3T Siemens Skyra Scanner (Erlangen, Germany). Diffusion‐weighted imaging was performed at ten b‐values *b* = [0, 10, 20, 30, 50, 70, 100, 200, 300, 500, 600] s.mm^−2^ and echo time (*T*_E_) = 72 ms. A spin‐echo *T*_2_ relaxometry acquired at *b*‐value = 0 s.mm^−2^ and at ten echo times, *T_E_* = [81, 90, 96, 120, 150, 180, 210, 240, 270, 300] ms. In addition, data acquired at *b*‐values 50 and 200 for *T_E_* = [81, 90, 120, 150, 180, 210, 240] ms. Voxel size was 0.9 × 0.9 × 2.5 mm^3^, reconstructed matrix 308 × 384 and 26 slices. Diffusion and relaxation measurements were obtained using a pulsed gradient spin‐echo with echo‐planar imaging (EPI) readout. DTI was acquired in 30 non‐colinear directions at b‐values 50 and 100 s.mm^−2^ and TE 63s and 69 ms respectively. DTI voxel size was 3.6 × 3.6 × 2.5 mm^3^, reconstructed matrix 78 × 96 and 36 slices.^[^ ^44^ ^]^

### Estimation of Fetal and Brain Weight from MRI Volumetry

A 3D steady‐state free procession of the uterus (*T*_E_ = 1.6 ms; repetition time [*TR*] = 3.38 ms; slice thickness = 2 mm; number of slices = 100–120; number of averages = 1; matrix size = 272 × 272; field of view (FOV) = 430 × 430 mm; average acquisition time ≈9 min) was acquired and segmented using ITK‐SNAP (version 3.8) to measure fetal volume.^[^ ^71^ ^]^ Overall fetal volume and brain volume were used to estimate both fetal weight and brain weight using a previously described conversion factor.^[^ ^72^ ^]^

### Blood Gas Measurements

Efforts to accurately characterize the relationship between *T*_2_ and SO_2_ in whole blood had led to the experimental and theoretical parameterization of the Luz–Meiboom model^[^ ^73^ ^]^ with varying degrees of model complexity.^[^ ^74^, ^75^ ^]^

A commonly used and simplified model, proposed by Wright et al.^[^ ^23^ ^]^ could be written as:

(1) $$\frac{1}{T_{2}}=\frac{1}{T_{2,0}}+K_{0}\left(1-\frac{SO_{2}}{100\%}\right)+K_{1}{\left(1-\frac{SO_{2}}{100\%}\right)}^{2}$$

where *T*_2_ is the measured *T*_2_ value of partially oxygenated blood, *T*_2,0_ is the *T*_2_ value of fully oxygenated blood, which is either assumed or measured in vitro, *K*_0_ and *K*_1_ are calibration factors that depend on field strength, average exchange time of water in blood *τ_𝑒𝑥_*, refocusing pulse interval *τ*_180_ and the specific readout technique. *T*_2,0_, *K*_0_ and *K*_1_ were held fixed at literature values of 148.4 ms, 1.4 s^−1^, and 104.4 s^−1^, respectively.^[^ ^22^ ^]^

### Placentome MRI Signal Model

**Figure 9:**
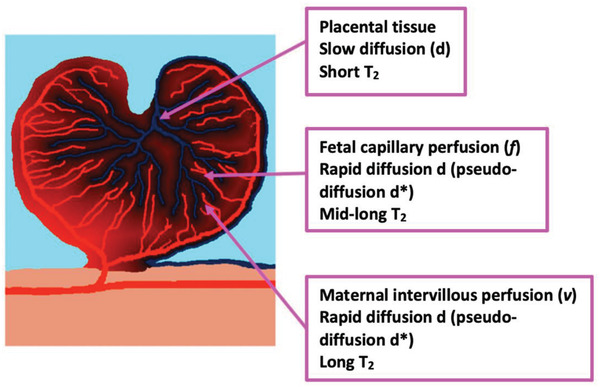
A representative drawing of the sheep placenta structure illustrating the division of the sheep placentome into three compartments and their respective MRI properties. Diffusivity (*d*) links to the overall cellularity of the tissue and serves as a convenient measure of the magnitude of diffusion. The *T*_2_ relaxation time of blood is positively associated with its oxygen saturation for a given hematocrit due to paramagnetic effect of deoxyhemoglobin.^[^ ^18^, ^27^ ^]^

A sheep‐specific signal model (**Figure** **9**) based on a previously used multi‐compartment MRI model of human placental perfusion was applied.^[^ ^15^ ^]^ The model consisted of three compartments: a fetal circulatory compartment, a maternal circulatory compartment, and a placental tissue compartment. In the pregnant sheep, both the fetal and maternal circulations were intra‐capillary, in contrast to the human placenta where maternal blood accumulated in the intervillous space and only the fetal circulation remained intra‐capillary, necessitating a sheep‐specific adaptation. *T*_2_ relaxation was positively associated with oxygen saturation for a given hematocrit due to the paramagnetic effect of deoxyhemoglobin causing stronger signal dephasing. Here it was assumed that the maternal circulation was highly saturated with a high *T*_2_ associated with the delivery of highly oxygenated blood, whilst the fetal circulation had a low (but unknown) *T*_2_.^[^ ^15^ ^]^ The remaining tissue was cell dense with low diffusivity and low *T*_2_. The sheep signal model was of the form:

(2) $$S\left(b,T_{E}\right)=S_{0}\left[e^{-bd*}\left(fe^{-T_{E}R_{2}^{f_{b}}}+ve^{-T_{E}R_{2}^{\mathrm{mb}}}\right)+\left(1-f-v\right)e^{-bd-T_{E}R_{2}^{\mathrm{ts}}}\right]$$

where *S* is the measured MR signal and *S*_0_ is the signal with no diffusion weighting (i.e., *b* = 0). The five independent model parameters were the feto‐placental blood volume fraction *f*, trophoblast apparent diffusivity *d*, which is the diffusion of water molecules in a cellular environment where the more cells present, the lower the diffusion of water molecules, pseudo‐diffusivity d∗, feto‐placental blood relaxation $R_{2}^{f_{b}}=1/T_{2}^{f_{b}}$ and maternal blood volume fraction *v*. Intra‐capillary fetal blood has rapid *d*, high d∗, and mid‐long $T_{2}^{f_{b}}$. Maternal blood with volume fraction *v* is in the intervillous space and has rapid *d*, high d∗, and long *T*_2_. The sheep signal model assumed that d∗ in fetal blood and maternal blood cannot be distinguished, although theoretically, the intra‐capillary flow properties might differ between the separate fetal and maternal circulations. Literature‐based values were used for highly‐saturated maternal blood relaxation $R_{2}^{mb}$ and tissue relaxation $R_{2}^{ts}$ at 3T of (150 ms)^−1^ and (42 ms)^−1^.^[^ ^76^, ^77^ ^]^ Feto‐placental SO_2_ could be estimated by solving Equation (1) given the feto‐placental *T*_2_ value, $T_{2}^{f_{b}}$ fitted in Equation (2).

### Image Processing

The placentome regions of interest (ROI) were manually segmented from the first *b* = 0 image (ITK‐SNAP Version 3.6.0, 2017) and double‐checked by a member of the team familiar with post‐mortem tissue analysis.^[^ ^44^ ^]^ The ROIs covered a representative area of each placentome, and ROIs were placed away from edges such that any residual movement artifact would not cause it to move out of the placentome. To reduce motion artifact, a rigid registration^[^ ^78^ ^]^ was applied followed by a nonrigid free‐form registration.^[^ ^24^ ^]^

### Model Fitting

Voxel‐by‐voxel model fitting was performed with a Levenberg–Marquardt algorithm applied to Equation (2) using inhouse software developed in MATLAB (The MathWorks, Natick, MA). The fitting routine was initialized with parameter estimates from model‐fitting results obtained from average placental ROI signal curves as in Melbourne et al.^[^ ^15^ ^]^ To stabilize the fitting when computing voxelwise estimates, the following constraints were chosen: 0 < *f* < 1 (no units), 0 < *d* < 1 (mm^2^s^−1^), 0 < *d* <1 (mm^2^ s^−1^), 0 < $T_{2}^{f_{b}}$ < 150 and 0 < *𝑣 <* 1 (no units).

### Human Data

The study was approved by the UK National Research Ethics Service and all participants gave written informed consent (London–Hampstead Research Ethics Committee, REC reference 15/LO/1488). Fourteen women beyond 24 weeks gestational age with uncomplicated pregnancies and twelve women with pregnancies complicated by early‐onset FGR gave informed consent to participate (see Figure 8). Early‐onset FGR was defined according to a Delphi consensus, as an estimated fetal weight < 10^th^ centile,^[^ ^79^ ^]^ with uterine or umbilical artery Doppler pulsatility index > 95^th^ centile or estimated fetal weight < 3^rd^ centile with or without Doppler ultrasound abnormality before 32 weeks of gestational age.^[^ ^80^, ^81^ ^]^ The control group was defined as women whose fetus had an ultrasound estimated fetal weight > 10^th^ centile. This data had been previously presented in^[^ ^27^ ^]^ but here this data was re‐analyzed for comparison with the data from sheep pregnancies.

### Estimation of Growth Rates in Sheep and Human Pregnancy

Growth rates from sheep pregnancy were defined using the birthweight and the weight estimated from MRI at 100 days gestation. The growth rate was thus defined as the difference in birthweight and estimated fetal weight from MRI (in grams) divided by the time interval in weeks. Relative brain volume was defined using the MRI segmentation of the fetal brain and whole fetal body. Estimated fetal density *ρ* was estimated at a value of 1.04.^[^ ^72^ ^]^

Growth rates from human pregnancy were defined using the estimated fetal weight from ultrasound at the time of MRI and the birthweight. The growth rate was thus derived from the difference in birthweight and estimated weight from ultrasound (Using the Hadlock formula to yield measurement in grams) divided by the time interval in weeks. Relative brain volume (RBV) was defined using the ultrasound estimate of head‐circumference, HC, and estimated fetal weight, EFW, using the formula: RBV = *ρ* HC^3^ / (6000 *π* ^2^ EFW).

### Statistical Analysis

The statistical analysis was performed using RStudio (version 4.0.3, 2020) and GraphPad Prism (version 9.2.0, 2021). Normality was assessed with the Shapiro–Wilk test using RStudio. Two‐way ANOVA was performed to examine the effect of group and gestational age on MRI parameters. Linear regression was performed using GraphPad Prism to compare the relationship between the MRI measurements of feto‐placental oxygenation against the reference fetal DAo oxygenation measured by catheterization. The systematic bias and limits of agreement between the two methods were evaluated by the Bland–Altman method. The correlations between recorded MRI measurements of feto‐placental SO_2_ and the reference DAo were assessed using Pearson's correlation coefficient (*r*). One‐way ANOVA with a post‐hoc Tukey HSD test was performed in RStudio to examine if MRI feto‐placental SO_2_ was predictive of growth rate in sheep and human fetuses. Numerical results were expressed as mean ± standard deviation (*sd*). Data were considered statistically significant when *p*‐values were less than 0.05.

## Conflict of Interest

The authors declare no conflict of interest.

## Data Availability

The data that support the findings of this study are available from the corresponding author upon reasonable request.
